# Discrimination between schizophrenia and other psychotic conditions by clinician’s difficulty in attunement: a reappraisal of the Praecox Feeling concept

**DOI:** 10.3389/fpsyg.2025.1534377

**Published:** 2025-03-17

**Authors:** Laura Fonzi, Mauro Pallagrosi, Cristiano Carlone, Angelo Picardi

**Affiliations:** ^1^Reverie Psychiatric Residential Facility, Rome, Italy; ^2^Community Service for Prevention and Early Intervention in Mental Health, Rome 1 Local Health Unit, Rome, Italy; ^3^San Giovanni-Addolorata Hospital, Acute Psychiatric Inpatient Unit, Rome 2 Local Health Unit, Rome, Italy; ^4^Centre for Behavioural Sciences and Mental Health, Italian National Institute of Health, Rome, Italy

**Keywords:** praecox feeling, schizophrenia, ACSE, empathy, psychopathology, intersubjectivity, psychiatric diagnosis

## Abstract

**Introduction:**

In the 1940s, Henricus Cornelius Rümke introduced the concept of *Praecox Feeling* (PF), a multifaceted clinician’s intuition about the nuclear essence of schizophrenia that may play a role in the diagnostic process. Many classical and contemporary psychopathologists have devoted attention to this concept and the issue of intuitive diagnosis of schizophrenia. However, so far very little empirical research was carried out on this topic. This study aimed at testing the hypothesis that the empathic failure described by Rümke as a major experiential dimension underlying the PF as measured by the ACSE Difficulty in Attunement scale can discriminate between schizophrenia and the other psychotic conditions.

**Methods:**

The study involved 49 clinicians and 326 patients (schizophrenia *N* = 161, schizoaffective disorder *N* = 47, delusional disorder *N* = 35, psychotic mood disorder *N* = 83) in several psychiatric inpatient and outpatient units. When they saw a new patient, the clinicians completed the Assessment of Clinician’s Subjective Experience questionnaire (ACSE) and the 24-item Brief Psychiatric Rating Scale (BPRS).

**Results:**

While no significant finding was observed in outpatients, several significant between-group differences in ACSE scores were found in inpatients. In multivariate analysis controlling for patient’s sex, age, educational level, and clinical severity as measured by BPRS total score, we found that clinicians reported higher levels of Impotence with patients affected by schizoaffective disorder and schizophrenia than with patients affected by psychotic mood disorder, and that clinicians reported higher levels of Difficulty in Attunement with patients affected by schizophrenia than with patients affected by delusional disorder and psychotic mood disorder.

**Discussion:**

Although our findings should be interpreted with caution due some study limitations, they corroborate the notion that the clinician’s feelings, and in particular empathic attunement and its disruptions, play a role in the diagnosis of schizophrenia. They provide preliminary support for Rümke’s hypothesis that the PF may help distinguishing between clinically overlapping psychotic conditions. Overall, this study highlights the importance for psychiatry to embrace the relational dimension of the clinical encounter, and to recognize the value of the clinician’s subjective participation within the clinical relationship itself.

## 1 Introduction

*It is remarkable that is rare for a diagnostician to be able to indicate exactly how he arrives at a diagnosis of schizophrenia*. With these exact words Henricus Cornelius Rümke, eminent Dutch psychiatrist, introduced in the 1940s the concept of *Praecox Feeling* (PF), a multifaceted clinician’s intuition about the nuclear essence of schizophrenia and a fundamental resource for the diagnostic process (Rümke, 1941; Rümke and Neeleman, 1990).

PF can be rightly considered a milestone for the formalization of the phenomenon of directly ‘sensing’ a psychopathological *gestalt*, and as such it has become over the years a prototype of the subjective-intuitive approach to diagnostic reasoning, often presented in opposition to the objective-operational one (Varga, 2013; Galbusera and Fellin, 2014; Gupta et al., 2019; Haliday, 2024). The popularity enjoyed by this concept is probably due to the author’s outstanding ability in distilling a number of substantial epistemological considerations in a six-pages keen and synthetic essay, which perspicuously addressed both theoretical issues and clinical practice.

Basically, Rümke posited that an expert psychiatrist, even before or without investigating the single psychopathological cues, can *experience* - through an immediate perceptive act - the specific schizophrenic coloring of a clinical picture. In other words, he postulated that a peculiar and distinguishable feeling, which rapidly emerges during the encounter and is specifically induced by the patient, decisively guides a receptive clinician towards a diagnosis of schizophrenia.

A careful examination of Rümke’s words suggests that this sort of interpersonal insight reflects two main experiential dimensions on the part of the clinician (Pallagrosi and Fonzi, 2018). On the one hand, the psychiatrist, *like any human being*, immediately feels the extraneity, the ‘intersubjective gap’ that divides her from the alienated mode of being of the schizophrenic person. The latter’s impaired capacity of emotionally tuning in and sharing a common experiential ground with the clinician – or, in Rümke’s words, the lack of a ‘rapprochement instinct’ – hinders the potential of empathic engagement, in a way that the clinician perceives as an internal malaise. On the other hand, the psychiatrist, *as an expert connoisseur* of the typical schizophrenic *gestalt* (e.g., psychomotor attitude, thought expression), implicitly uses the ability to rapidly and synthetically capture a number of subtle alterations, and assigns them diagnostic significance even before a reflective assessment. In other words, as a skilled observer, he directly recognizes the whole aspect of the schizophrenic patient, in the same way as each of us immediately recognizes a familiar object, such as a table or a bus, without examining its individual elements (Schwartz and Wiggins, 1987).

This dual level of the PF can be also identified in the two main lines of thought developed by classical and contemporary psychopathologists who, like Rümke, have dealt with the issue of intuitive diagnosis of schizophrenia. A considerable number of scholars, in fact, devoted particular attention to the intersubjective, empathic pole of the PF, and underscored the role of the peculiar resonance of the schizophrenic “being-with” in generating such a pathognomonic atmosphere (Buonarroti et al., 2022). Several classical authors thoroughly described the schizophrenic mode of experiencing oneself, the world and the others, with all its lacks and impasses, and outlined traditional concepts such as *autism*, *obliqueness*, *lack of vital contact*, or *loss of natural evidence* (Minkowski, 1927; Binswanger, 1956; Blankenburg, 1971). Contemporary psychopathologists have later refined these phenomenological accounts, and introduced the idea of basic self-disorders in schizophrenia, which reverberate on both psychopathological expressivity and interpersonal hindrance. By providing these theoretical refinements, they have further accounted for the feeling of gasp and the perception of common sense collapse experienced by clinicians engaged with schizophrenic patients (Parnas, 2011; Fuchs, 2015; Sass and Feyaerts, 2024).

The concept of self-disorder and disrupted self-other interaction as the core of schizophrenic pathology—and, more broadly, of severe psychiatric disorders—has also become a central focus for neuroscience researchers, who are increasingly interested in investigating the nuances of neural phenomena such as mirroring, reciprocity, and communication (Schilbach, 2016; Aragona, 2022; Schilbach and Redcay, 2025).

On the other hand, another group of scholars has focused on the place of the PF in the epistemic path, and analyzed how this intuitive phenomenon integrates the diagnostic reasoning. Following the argument of its rapid emerging during the encounter, these authors highlighted how the PF, due to its pre-verbal and non-mediated nature, actually comes up as the first step of the interpersonal knowing process, and therefore of the psychopathological understanding (Schwartz and Wiggins, 1987; Gupta et al., 2019; Gozé, 2022). As such, the PF, as a paradigm of *gestaltic* diagnostic impressions, has the potential both to effectively identify significant, even when subtle, clinical phenomena, and to guide the explicit assessment and support the diagnostic process, especially when other diagnostic cues are few or non-specific (e.g., at the onset of the disease or in paucisymptomatic conditions). Clearly, as claimed by Rümke himself, since the evaluation entails different and stratified interpersonal levels (first-, second-and third-personal), the PF should be intended only as a part of the whole diagnostic process, to which detached observation, analytic investigation, and operational grids are equally relevant (Fuchs and Dalpane, 2022; Zaninotto and Altobrando, 2022).

According to Rümke, the phenomenon of the PF basically concerns all psychiatrists, and in recent years epidemiological surveys confirmed that still today many clinicians acknowledge using this tool in their everyday practice (Moskalewicz and Gozé, 2022). For this reason, it is remarkable that so far very little empirical research was carried out on this topic. Indeed, apart from a few pioneer attempts with inconsistent methodologies and substantial limitations (Grube, 2006; Dimic et al., 2010; Ungvari et al., 2010), an *in vivo* analysis of the emergence of specific subjective feelings in the clinician and of their relation with the final diagnostic judgment has been lacking.

In 2014, our group published the validation study of a new psychometric instrument, named *Assessment of Clinician’s Subjective Experience* (ACSE) (Pallagrosi et al., 2014), which included Rümke and his concept of the PF among its major sources of inspiration. The ACSE is a short questionnaire, easy to complete and to score, which does not require specific training and can be used with any kind of patient and by any psychiatrist, independent of years of clinical experience. It provides a valid, overall representation of the clinician’s subjective experience during the first assessment of a mentally disordered person. This experience is characterized by five empirically-derived dimensions, namely Tension, Difficulty in Attunement, Engagement, Disconfirmation, and Impotence.

The ACSE makes it possible to investigate a wide range of research topics, including those related to the value of the clinician’s subjective impression in specific diagnostic situations. Indeed, within naturalistic settings, the ACSE was found to yield different clinician’s subjective experience profiles in relation to the encounter with patients belonging to different diagnostic categories, in a way that is mostly consistent with theoretical hypotheses and everyday accounts (Pallagrosi et al., 2016; Pallagrosi et al., 2022). A peculiar connection was found between schizophrenia and Difficulty in Attunement, as the clinician’s level of this dimension was found to be significantly higher when interacting with patients diagnosed with schizophrenia than with patients suffering from a manic or mixed bipolar episode, cluster B personality disorder, and depressive or anxiety disorders. This finding remained significant when controlling for clinical severity, and was subsequently corroborated by a large study that identified the psychotic psychopathological dimensions (i.e., positive and negative symptoms) as the strongest predictors of clinician’s Difficulty in Attunement (Picardi et al., 2017).

The Difficulty in Attunement dimension basically refers to a specific struggle in establishing human contact with the patient and in identifying with her mode of experiencing, mostly accompanied by a variable degree of feeling of extraneity. Given its item composition and its empirical relation with schizophrenia and psychotic dimensions, it is reasonable to relate this experiential domain to the nuclear empathic impasse around which an intuitive judgment such as the PF develops (Fonzi et al., 2022). In fact, Difficulty in Attunement shares with the experience of the PF not only these characteristics, but also two other notable aspects. First, in all our studies the mean scores on Difficulty in Attunement did not differ between experienced and young psychiatrists, independent of other clinical or setting variables. This finding suggests that such an experience is mostly not affected by technical expertise, in the same way as the empathic nucleus of the PF seems not to be grounded on a trained ability, but on a basic human one. Rather, it is the clinical use of this kind of perception that discriminates between a naïve clinician and an expert one. Second, Difficulty in Attunement was found not to be substantially affected by ethnocultural differences between clinicians and patients (Fonzi et al., 2020).This finding further corroborates the idea that this dimension refers to an innate inter-human phenomenon, basically free of individual or collective superstructures, as the basic empathic act is theoretically conceived by many phenomenologists (Gallagher and Zahavi, 2012; Ratcliffe, 2012; Stanghellini, 2016). Taken together, these findings suggest that only a profound disturbance of the patient’s self is able to critically impair the implicit and natural attunement between him and the clinician.

In the light of the many links between the ACSE Difficulty in Attunement dimension and the PF, we planned to corroborate and extend our previous findings through a new study specifically focused on the relationship between the ACSE dimensions and all the main psychotic disorders. The study aims at testing the hypothesis that the Difficulty in Attunement dimension is able to discriminate between schizophrenia and the other psychotic conditions, as assumed by Rümke for the PF. The findings of this study may help clarifying whether through Difficulty in Attunement it is possible to achieve more reliable observations about the actual role of the PF in the diagnostic process. They may also broaden our reflection about the PF and possibly contribute to reintroduce this concept into the epistemological and the nosological debate.

## 2 Materials and methods

### 2.1 Setting and participants

The study was carried out in a number of psychiatric inpatient and outpatient units in Rome, Italy. The clinicians working in these units were asked to include in the study all previously unknown psychotic patients that they met for clinical and diagnostic evaluation. To be included in the study, a patient had to meet the following criteria: (1) age of 18 years or more; (2) Italian nationality (to rule out potential problems in mutual understanding due to language difficulties in foreign patients); (3) absence of intellectual disability or significant cognitive impairment; (4) absence of substance use disorder; (5) absence of major medical illness; (6) diagnosis of Schizophrenia, Schizoaffective Disorder, Delusional disorder, Major Depressive Disorder with psychotic features, or Bipolar Disorder with psychotic features.

Overall, 33 psychiatrists and 16 senior psychiatry residents with different theoretical backgrounds and attitudes were involved in the study. The mean number of patients rated per clinician was 6.65 (range 1–42). The clinicians’ characteristics are reported in Table 1. They recruited a total of 326 psychotic patients, of whom 20.2% were seen in outpatient clinics, and 79.8% in inpatient settings (emergency department, general hospital psychiatric ward, or private psychiatric inpatient facility). The mean duration of the examination was 36.0 ± 14.4 min. Patients’ demographic and clinical characteristics are summarized in Table 2.

**Table 1:** Clinicians’ characteristics.

	Psychiatrists (*N* = 33)		Senior psychiatry residents (*N* = 16)	
Dependent variable	*N* (%)	Mean ± SD	*N* (%)	Mean ± SD
Sex				
Male	15 (45.5)		5 (31.2)	
Female	18 (54.5)		11 (68.8)	
Age		40.0 ± 8.5		30.5 ± 2.1
Years of post-residency experience		8.9 ± 8.1		
Theoretical background				
Psychodynamic theory	12 (36.4)		10 (62.5)	
Clinical/biological psychiatry	12 (36.4)		4 (25.0)	
Cognitive-behavioral theory	4 (12.1)		0	
Phenomenology	3 (9.1)		2 (12.5)	
Family systems theory	2 (6.1)		0	

**Table 2:** Patients’ characteristics.

Dependent variable	*N* (%)	Mean ± SD
Sex		
Male	165 (50.6)	
Female	161 (49.4)	
Age		45.1 ± 13.3
Marital status		
Unmarried	230 (70.6)	
Married	48 (14,7)	
Separated	14 (4.3)	
Divorced	17 (5.2)	
Widowed	7 (2.1)	
Missing information	10 (3.0)	
Education		
No formal education	6 (1.8)	
Primary school	12 (3.7)	
Junior high school	77 (23.6)	
Senior high school	139 (42.6)	
Bachelor degree	14 (4.3)	
Master degree	56 (17.2)	
Missing information	22 (6.7)	
Clinical setting		
Outpatient clinic	66 (20.2)	
Inpatient setting (emergency department, general hospital psychiatric ward, private psychiatric inpatient facility)	260 (79.8)	
Primary axis I diagnosis		
Schizophrenia	161 (49.4)	
Schizoaffective disorder	47 (14.4)	
Delusional disorder	35 (10.7)	
Unipolar depression with psychotic features	18 (5.5)	
Bipolar disorder, manic or mixed episode with psychotic features	54 (16.6)	
Bipolar disorder, depressive episode with psychotic features	11 (3.4)	
BPRS total score		61.5 ± 14.9

According to the Italian legislation, purely observational, cross-sectional studies based on data collected as part of routine patient assessment do not need formal ethical approval. The clinicians were the study subjects and provided their informed consent to take part in the study. The study did not involve any risk or discomfort for participants.

### 2.2 Assessment

A standardized form was used to gather information about demographic variables, setting, duration of the evaluation, and clinical diagnosis according to DSM-IV-TR (APA, 2000) or ICD-10 (World Health Organization, 1992) criteria.

Immediately after seeing the patient, the clinician completed the Assessment of Clinician’s Subjective Experience (ACSE) and the 24-item Brief Psychiatric Rating Scale (BPRS).

The ACSE is a self-completed instrument that was specifically developed to measure clinicians’ subjective experience during the interaction with patients. It consists of 46 items, each rated on a 5-point scale ranging from 0 to 4. The instrument yields scores on five scales, named Tension, Difficulty in Attunement, Engagement, Disconfirmation, and Impotence. Studies on both adult (Pallagrosi et al., 2014) and adolescent (Picardi et al., 2021) patients provided robust evidence of validity and reliability for the ACSE. The Tension scale consists of items indicating physical tension and clumsiness, reduced spontaneity, and feelings of worry, nervousness, and alarm (e.g., ‘I felt tense in moments of silence’, ‘I maintained a rigid posture’, ‘I was afraid that the patient could act unpredictably’); greater scores indicate higher tension during the visit. The Engagement scale includes items describing the degree of the psychiatrist’s involvement with the patient, such as feelings of boredom, indifference, detachment, lack of attention and, conversely, desire to take care of the patient, and feelings of involvement in the patient-physician relationship, emotional closeness, and tenderness (e.g., ‘I experienced a feeling of tenderness towards the patient’, ‘I felt emotionally close to the patient’). Differently from the items indicating closeness to the patient, the items covering detachment from the patient are reverse-keyed, so that higher scores on this scale indicate greater involvement with the patient. The Disconfirmation scale consists of items describing a failure to establish an authentic relationship with the patient, and feelings of being manipulated, rejected, criticized or devalued by the patient (e.g., ‘I felt depreciated by the patient’, ‘I felt judged by the patient’, ‘I felt rejected by the patient’, ‘I felt that I did not exist for the patient’.); higher scores reflect greater feelings of disconfirmation. The Impotence scale contains items indicating feelings of helplessness, frustration, desolation, emptiness, loneliness, and being drained (e.g., ‘I felt a sense of loneliness’, ‘I felt a sense of emptiness’, ‘At the end of the interview I felt a sense of impotence’); higher scores indicate greater feelings of impotence.

The Difficulty in Attunement scale contains items describing difficulty in establishing emotional contact, being empathic, understanding the patient’s experience, and communicating with the patient. Given the relevance of this scale for the present study, we report here all its items: ‘At the beginning of the interview I struggled to establish an emotional connection with the patient’; ‘I found it difficult to follow the train of thoughts expressed by the patient’; ‘I perceived a discordance between the way in which the patient experienced some of his/her life events and the way in which I would have experienced them’; ‘I simplified my communication by modifying my usual language’; ‘I carefully chose my words in order not to scare the patient’; ‘I carefully chose my words in order to be easily understood by the patient’; ‘I tempered the tone of my voice in relation to the patient’s state’; ‘There were times when I felt the way in which the patient gave sense to his/her own experiences was alien to me’; ‘I had difficulties in identifying myself with the patient’; ‘I felt a sense of alienation from the patient’. Higher scores reflect greater difficulties in attunement to the patient.

The 24-item BPRS (Lukoff et al., 1986; Ventura et al., 1993) is a clinician-rated instrument, which is an expanded standardized version of the original 16-and 18-item versions of the BPRS (Overall and Gorham, 1962; Overall, 1974). The items are scored on a 7-point severity scale. We used the 0–6 scoring system, so that the total score ranges from 0 to 144. Higher scores indicate greater severity of psychiatric symptoms. We used a validated Italian version (Morosini et al., 1995) which is based on the BPRS manual of administration (Ventura et al., 1993) with defined anchor points and detailed probe questions and rules for scoring.

### 2.3 Statistical analysis

All statistical analyses were performed using SPSS for Mac, version 29.0. All tests were two tailed, with alpha set at 5%. First, demographic and clinical characteristics were summarized using appropriate descriptive statistics, i.e., mean and standard deviation or frequencies.

Subsequently, the Chi-Square test was used to test for differences between diagnostic groups in categorical (sex, education, setting) variables. Also, analysis of variance with Tukey-corrected post-hoc comparisons was used to test for differences between diagnostic groups in continuous (age, BPRS total score, ACSE scale scores) variables.

Finally, analysis of covariance (ANCOVA) was used to test for differences between diagnostic groups in scores on each ACSE scale that displayed a significant association with diagnosis in univariate analysis, while controlling for patient’s age, sex, education, and clinical severity as measured by BPRS total score. Prior to each analysis, ANCOVA assumptions were tested by inspecting the normal probability plot of residuals to assess normality, by examining the scatterplot of residuals versus predicted values to assess linearity, by performing Levene’s test as well as examining the scatterplot of residuals versus predicted values to assess homogeneity of variance, and by performing interaction tests to verify the homogeneity of regression slopes. Also, variable inflation factor (VIF) scores for each variable were used to assess the assumption of multicollinearity. Furthermore, Mahalanobis distance was computed to screen for multivariate outliers using a conservative criterion of *p* < 0.001. Each ANCOVA model included the ACSE scale under examination as dependent variable and patient’s age, sex, education level, and BPRS total score as independent variables. Patient’s sex was modeled as a dummy variable with women as the reference category. Post-hoc pairwise comparisons were adjusted for multiple testing with the Sidak method.

## 3 Results

### 3.1 Description of the diagnostic groups

As detailed in Table 3, as compared with the other groups, the patients with schizophrenia displayed a greater proportion of males and lower educational level. Also, they showed higher scores on the BPRS than patients with delusional disorder.

**Table 3:** Demographic and clinical characteristics by diagnostic group.

	Schizophrenia (*N* = 161)		Schizoaffective disorder (*N* = 47)		Delusional disorder (*N* = 35)		Mood episode with psychotic features (*N* = 83)	
Dependent variable	*N* (%)	Mean ± SD	*N* (%)	Mean ± SD	*N* (%)	Mean ± SD	*N* (%)	Mean ± SD
Sex§								
Male	98(60.9)		22 (46.8)		11 (31.4)		34 (41.0)	
Female	63 (39.1)		25 (53.2)		24 (68.6)		49 (59.0)	
Age		43.5 ± 12.7		44.3 ± 10.5		47.1 ± 11.7		47.8 ± 15.7
Education @								
Primary school or no formal education	9 (6.0)		2 (4.3)		0 (0.0)		7 (8.4)	
Junior high school	49 (30.4)		10 (21.3)		8 (22.9)		10 (12.0)	
Senior high school	69 (42.9)		21 (44.7)		10 (28.6)		39 (47.0)	
Bachelor degree	7 (4.3)		1 (2.1)		2 (5.7)		4 (4.8)	
Master degree	16 (9.9)		7 (14.9)		14 (40.0)		19 (22.6)	
Clinical setting								
Outpatient clinic	30 (18.6)		6 (12.8)		11 (31.4)		19 (22.9)	
Inpatient ward or emergency room	131(81.4)		41 (87.2)		24 (68.6)		64 (77.1)	
BPRS total score		62.9 ± 14.8#		59.6 ± 12.3		54.8 ± 16.7		62.6 ± 15.1#

§ *p* < 0.01 overall Chi square; no standardized residual greater than 1.96. @ *p* < 0.001 overall Chi square; standardized residual greater than 1.96 for master degree in Schizophrenia and Delusional disorder, and for junior high school in Mood episode with psychotic features. # Significantly higher than Delusional disorder (*p* < 0.05).

### 3.2 Between-group differences in ACSE scores

Mean ACSE scores by diagnostic group are summarized in Table 4. When considering both types of setting together, only a number of significant between-group differences in ACSE scores were observed. The clinicians reported higher levels of Difficulty in Attunement with patients with schizophrenia than with those affected by mood episode with psychotic features (*p* = 0.001) and delusional disorder (*p* < 0.05). Also, clinicians reported higher scores on Impotence with patients affected by schizoaffective disorder than with those affected by mood episode with psychotic features (*p* < 0.01) and delusional disorder (*p* < 0.05).

**Table 4:** ACSE scores by diagnostic group and setting.

Both outpatients and inpatients					Outpatients				Inpatients			
	Schizophrenia (*N* = 161)	Schizoaffective disorder (*N* = 47)	Delusional disorder (*N* = 35)	Mood episode with psychotic features (*N* = 83)	Schizophrenia (*N* = 30)	Schizoaffective disorder (*N* = 6)	Delusional disorder (*N* = 11)	Mood episode with psychotic features (*N* = 19)	Schizophrenia (*N* = 131)	Schizoaffective disorder (*N* = 41)	Delusional disorder (*N* = 24)	Mood episode with psychotic features (*N* = 64)
Tension	9.6 ± 6.6	11.4 ± 8.0	8.0 ± 6.8	8.7 ± 6.8	9.1 ± 7.4	9.2 ± 5.5	8.6 ± 7.1	10.0 ± 6.2	9.7 ± 6.4	11.7 ± 8.4	7.7 ± 6.8	8.3 ± 7.0
Difficulty in attunement	22.0 ± 7.5#	21.2 ± 6.8	18.4 ± 7.7	18.4 ± 6.5	19.4 ± 6.5	20.5 ± 4.7	20.4 ± 5.5	18.4 ± 5.2	22.6 ± 7.6@	21.3 ± 7.1	17.5 ± 6.8	18.4 ± 6.8
Engagement	17.7 ± 3.9	17.2 ± 4.3	18.1 ± 5.0	18.1 ± 4.7	17.9 ± 4.3	16.2 ± 5.9	18.8 ± 5.7	19.6 ± 5.5	17.7 ± 3.8	17.4 ± 4.3	17.8 ± 4.8	17.7 ± 4.5
Disconfirmation	7.5 ± 5.1	9.4 ± 5.5	8.2 ± 6.9	8.3 ± 5.8	6.5 ± 4.9	8.5 ± 3.7	7.0 ± 7.7	6.8 ± 4.9	7.7 ± 5.1	9.6 ± 5.8	8.7 ± 6.6	8.7 ± 6.1
Impotence	10.9 ± 6.8	13.0 ± 7.2§	8.9 ± 6.4	8.7 ± 5.6	9.1 ± 5.5	13.8 ± 6.7	10.0 ± 8.4	11.0 ± 5.5	11.3 ± 7.0^	12.9 ± 7.3$	8.4 ± 5.3	8.1 ± 5.4

# Significantly higher than Mood episode with psychotic features (*p* = 0.001) and Delusional disorder (*p* < 0.05). § Significantly higher than Mood episode with psychotic features (*p* < 0.01) and Delusional disorder (*p* < 0.05). @ Significantly higher than Delusional disorder (*p* = 0.01) and Mood episode with psychotic features (*p* = 0.001). ^ Significantly higher than Mood episode with psychotic features (*p* < 0.01). $ Significantly higher than Delusional disorder (*p* < 0.05) and Mood episode with psychotic features (*p* < 0.01).

More pronounced between-group differences were observed when the analyses were performed separately for outpatients and inpatients. While no significant finding was observed in outpatients, several significant between-group differences in ACSE scores were found in inpatients. Clinicians reported lower levels of Impotence with patients affected by mood episode with psychotic features than with patients affected by schizophrenia and schizoaffective disorder (both *p* < 0.01). Also, they reported lower levels of Impotence with patients affected by delusional disorder than with those affected by schizoaffective disorder (*p* < 0.05). The most prominent between-group differences were observed in Difficulty in Attunement. In fact, clinicians reported higher levels of Difficulty in Attunement with patients affected by schizophrenia than with patients affected by delusional disorder (*p* < 0.01) and psychotic mood episode with psychotic features (*p* = 0.001).

Given that univariate analysis showed that the diagnostic groups differed on severity of symptoms and a number of demographic variables, as described in the Methods section we performed multivariate analysis on the inpatient subgroup in order to control for potential confounders. Two ANCOVA models were built, the first including Impotence as the dependent variable, and the second including Difficulty in Attunement as the dependent variable. In both cases, no multivariate outliers were found. These models included 242 and 243 patients with complete data for all variables, respectively.

In the first model, after adjustment by covariates, Impotence varied significantly with diagnostic group, with *F*(3, 234) = 6.56, *p* < 0.001, partial eta squared = 0.078. Post-hoc comparisons showed that clinicians reported higher levels of Impotence with patients affected by schizoaffective disorder (*p* = 0.002) and by schizophrenia (*p* = 0.003) than with patients affected by psychotic mood disorder. Among the covariates, only clinical severity as measured by BPRS total score was significantly associated with scores on Impotence, with *F*(1, 234) = 5.68, *p* = 0.018, partial eta squared = 0.024.

In the second model, after adjustment by covariates, Difficulty in Attunement varied significantly with diagnostic group, with *F*(3, 235) = 6.80, *p* < 0.001, partial eta squared = 0.080. *Post-hoc* comparisons showed that clinicians reported higher levels of Difficulty in Attunement with patients affected by schizophrenia than with patients affected by delusional disorder (*p* < =0.036) and psychotic mood disorder (*p* < 0.001). Among the covariates, only clinical severity as measured by BPRS total score was significantly associated with scores on Difficulty in Attunement, with *F*(1, 235) = 53.16, *p* < 0.001, partial eta squared = 0.184.

## 4 Discussion

We have already quoted Rümke’s words about the tools psychiatrists rely on to formulate a diagnosis of schizophrenia. In his opinion, as well as in the opinion of other authors who delved into the same subject, intuitive and subjective phenomena can significantly shape the diagnostic judgment of experienced clinicians. The most accredited interpretation for this phenomenon is based on seminal contributions about the intersubjective space between the clinician and the patient, which identified empathic stumbles and radical dissonances as near-specific markers of the encounter with the schizophrenic mode of being-with (Parnas, 2011; Varga, 2013; Fuchs, 2015).

Epidemiological observations suggest that intuitive judgments such as the *Praecox Feeling* are still implicitly ingrained in the everyday practice of most psychiatrists (Moskalewicz and Gozé, 2022).This finding strongly supports the recent call from prominent scholars who urged a renewed attention to the subjective experience of both patient and clinician. For decades, indeed, psychiatry has been largely driven by biological research, rooted in the theoretical premise that mental diseases should be classified and studied through objectifying and quantifiable methods. This approach has mostly relegated subjective experiences to the realm of idiosyncratic or confounding data. Yet, in clinical practice the idea that mental illness is grounded in a psyche that cannot be reduced to a collection of symptomatic behaviors has not been discarded, and most clinicians still acknowledge that the psychic world is primarily understood through the existential domain in which it unfolds, that is, the *Mitwelt* of being with others; in other words, the intersubjective domain.

This study aimed at reexamine the concept of PF by means of a quantitative assessment instrument that validly and reliably investigates the clinician’s subjective side of the clinical encounter, i.e., the ACSE. In particular, we examined 326 first diagnostic interviews with psychotic patients through the lens of the ACSE, in particular its Difficulty in Attunement dimension, which seems to be fairly representative of the empathic failure that lies at the heart of the PF.

We found that two ACSE dimensions, namely Impotence and Difficulty in Attunement, are able to discriminate between patients with different psychotic conditions. As far as the first is concerned, we found that clinicians reported lower levels of Impotence with inpatients affected by psychotic mood disorder as compared with inpatients affected by schizophrenia or schizoaffective disorder. This finding is consistent with our previous observation that perceived impotence is greater when seeing patients with mental disorders in which substantial improvement is notoriously more difficult to achieve and that tend to have a chronic course (Pallagrosi et al., 2016).

We found that the degree of Difficulty in Attunement is significantly greater when clinicians face patients with schizophrenia as compared to both delusional disorder or mood episodes with psychotic symptoms. This association remains significant also in multivariate analysis, i.e., it is independent of patient’s clinical severity, age, sex, and education level. Also, while this association is pronounced in inpatient settings, it tends to lose significance in outpatient consultations.

This finding is quite in line with Rümke’s hypothesis, and suggests that the disruption of empathic resonance in schizophrenia is not to be interpreted as a mere result of the patient’s psychopathological severity or symptomatic expressiveness. Rather, it represents the reflection of a profound disturbance of the patient’s experiential structure, i.e., the so-called *alienness* that specifically hinders the clinician’s possibility to find the *fellowman* into the patient, or–as Rümke used to say–to *find the patient within himself*.

This finding is also consistent with the common clinical observation that, although a struggle in attuning to the patient’s experience exists across all psychotic disorders, the empathic failure experienced in the relationship with schizophrenic patients is considerably different from that experienced with paranoid, manic, or depressed psychotic patients. Indeed, reality distortion and clinical severity being comparable, schizophrenic patients elude the clinician’s ability to *feel* and to *immediately* recognize them to a significantly greater extent as compared to patients with other psychotic disorders.

The specificity of this association between schizophrenia and difficulty in attunement is also indirectly supported by the lack of differences in other areas of the relational struggle, such as tension, sympathetic engagement, and feelings of disconfirmation. These experiences, indeed, despite being as distressing as difficulty in attunement, are less linked with the field of basic inter-human reciprocity.

It should be noted that no significant differences in scores on Difficulty in Attunement were observed between encounters with schizophrenic patients and those with patients affected by schizoaffective disorder. This finding, rather than being interpreted as evidence against the hypothesis of the uniqueness of empathic failure in schizophrenia, probably reflects the uncertainty surrounding schizoaffective disorder as a diagnostic entity. Indeed, although this diagnosis is commonly used in clinical practice, its validity is quite controversial. In the literature, it is a common finding that patients diagnosed as schizoaffective actually present themselves with significantly different phenotypic, developmental, neural, and genetic characteristics, and very few studies support the hypothesis that schizoaffective disorder is a separate disease, distinct from schizophrenia and mood disorders (Lake and Hurwitz, 2006). Also, the findings of a large prospective study that examined the relationship between the ratio of non affective psychosis to mood disturbance and long-term outcome cast further doubt on the validity of schizoaffective disorder and suggest that many patients diagnosed as schizoaffective actually belong to the schizophrenic spectrum (Kotov et al., 2013). Therefore, a reasonable explanation for the lack of significant differences in scores on Difficulty in Attunement between schizophrenia and schizoaffective disorder is due to the fact that the schizoaffective category actually included many patients with schizophrenia. Indeed, together with schizophrenia and schizotypal personality disorder, schizoaffective disorder is the diagnosis most commonly mentioned as part of the schizophrenia spectrum (Mamah and Barch, 2011).

A finding that invites a careful reflection is the observation that the clinician’s empathic discomfort is most pronounced in acute inpatient situations, whereas the distinction between the interactions with patients affected by schizophrenia and those diagnosed with other psychotic disorders tends to disappear in outpatient settings, where patients present with more stable conditions. In other words, it seems that the clinician’s experience of a challenging attunement is not solely promoted by the schizophrenic patient’s intersubjective core disturbance, but is also linked to the acute state of illness. This finding is particularly intriguing, as Rümke himself, in a later essay (Rümke, 1963), claimed that a downstream reorganization of the schizophrenic patient’s personality after the most acute phases of illness may allow the clinician to empathize with certain aspects of the patient. The author based this hypothesis right on his experience with chronic patients, who, after a long history of illness, appeared to him more *recognizable* or *closer* as compared to those still in the midst of the psychotic breakdown, to the point that they no longer elicited PF in the clinician.

It should be acknowledged that our study could not directly test this Rümke’s hypothesis, since we did not investigate the intersubjective experience with schizophrenic patients according to a diachronic perspective. However, the difference that we observed between acute and stable patients in terms of prominence of empathic failure encourages future studies correlating ACSE scores, particularly Difficulty in Attunement scores, with longitudinal data involving changes in the clinical phase.

A number of methodological limitations should be considered to properly contextualize our findings. First, the ACSE can measure only thoughts, feelings and behaviors of which the respondent is consciously aware, which is a limitation inherent in all self-report instruments. Therefore, our findings pertain only to conscious subjective experience. Also, the instrument can measure only those thoughts, feelings and behaviors that the respondent is able to recall. The completion of the instrument immediately after the encounter should have maximized the spontaneity and richness of the answers, while reducing any fall-off in recall.

Further, although the ACSE underwent an extensive, theoretically-based development process, it was empirically validated in both adult and adolescent psychiatric settings, and it covers a number of major aspects of subjective experience, it does not purport to measure every nuance of clinicians’ subjective experience. While it should be acknowledged that there might be some potentially important aspects of clinicians’ subjectivity that are not covered by the instrument, our findings suggest that the portion of subjectivity that is measured by the ACSE, however partial, can discriminate between schizophrenia and other psychotic conditions.

A third limitation concerns the diagnostic evaluation. Although diagnoses were informed by the most widely accepted nosographic classifications, they were not established through a highly reliable methodology, such as a standardized interview. This aspect may have allowed for greater influence from the psychiatrist’s personal interpretation, potentially limiting the reliability of the diagnoses themselves. Given that diagnosis is a critical variable in the study, this limitation may slightly affect the strength of the inferences that can be drawn from the study. Nonetheless, it is important to note that the majority of clinicians involved in the study were highly experienced, while the trainees were senior residents with at least 2 years of clinical practice. Moreover, their clinical evaluations were supplemented by a comprehensive psychopathological assessment, conducted using the BPRS alongside its coding manual. Therefore, while their final diagnostic judgments may not have been as consistently reproducible as those derived from an algorithmic, standardized approach, their accuracy was unlikely to be significantly compromised.

Another potential limitation relates to the independence of the diagnostic assessment. Specifically, the same psychiatrist who completed the ACSE was also responsible for making the diagnosis. This raises the concern that the diagnosis itself might have been influenced by the clinician’s own subjective experiences, that were measured by the ACSE. For instance, a psychiatrist experiencing a sense of alienness might be more likely to diagnose schizophrenia, which may lead to an overestimation of the relationship between this diagnosis and higher scores on Difficulty in Attunement. Although only an independent diagnostic evaluation by another clinician can completely rule out this possibility, it can likely be excluded that the reciprocal influence between subjective experience and diagnosis has been so strong to substantially distort the diagnostic judgment. The clinicians participating in the study were unaware of its specific aims, making it unlikely that they consciously adjusted their subjective experiences to align with their diagnostic decisions, or vice versa. Furthermore, because only the ACSE had to be completed immediately after the encounter with the patient, the clinicians had ample time to formulate their diagnoses and to integrate their initial impressions with more deliberate considerations such as clinical history, psychopathological assessment, and input from colleagues. As a result, the final diagnosis represented a balanced synthesis of intuitive response and reflective judgment, which should have minimized the influence of individual biases or idiosyncratic elements. Although the non-independence of assessments does not undermine the study’s main findings, further studies using standardized and independent diagnoses are needed to advance our understanding of the relationship between the clinician’s subjective experience and diagnosis.

A fifth limitation is the potential influence of unmeasured clinician-related variables, such as personal attitudes, personality traits, values, and preferences, which may act as confounding factors as they affect the intensity and mode of reaction towards others. Clinicians, after all, are individuals, and when assessed through a “subjective” lens, their personal characteristics may become apparent. However, it is important to emphasize that the ACSE is designed to measure state rather than trait phenomena, as it does not measure the clinician’s usual way of responding to others but rather captures changes in the emotional state of the clinician that occur specifically during the clinical encounter with a given patient. Moreover, for any unmeasured clinician variable to actually produce systematic bias, it would need to be differentially distributed across patient groups. Given the large sample size of patients and the diverse demographic and theoretical backgrounds of the participating clinicians, this scenario is unlikely.

In conclusion, while our findings should be interpreted with caution due to the limitations previously discussed, they generally support the belief held by many professionals that the clinician’s feelings, and in particular empathic attunement and its disruptions, have still a role in the diagnosis of schizophrenia. They further corroborate the Rümke’s hypothesis that the PF is a sensitive tool for distinguishing between clinically overlapping psychotic conditions, and may also undergo changes over time. In this sense, our study represents a significant advancement in both the understanding and the validation of the PF as a valuable integrative tool for diagnosis.

From this perspective, it should be noted that quite recently the research group led by Thomas Fuchs has called for a more thorough and specific empirical research on the PF, which they elaborated on in a rich and original essay (Vial et al., 2024). We fully agree with these authors about the need to explore more deeply a concept, as well as its corresponding phenomenon, that has long been neglected, oversimplified or misrepresented. More specifically, we were particularly struck by the authors’ articulation of the concepts of *intercorporeal diagnosis* and *close remoteness*, as they not only reflect the interpersonal dynamics of contact, but also highlight an embodied dimension of the PF that is explicitly covered by some items of the ACSE Difficulty in Attunement scale that inquire about gaze, tone of voice, and movements. The time seems truly ripe for a fruitful cooperation between phenomenological reflection and empirical research.

Finally, we further emphasize that the PF, as a paradigm of subjective and relational processes involved in understanding, synthetically captures the epistemological value of the clinical interaction. The evidence supporting its role in everyday assessment brings it back to the forefront of clinical reasoning, and reinforces the attention toward the relational dimension of the diagnostic process. This by no means implies that the standardized, third-person, or neurobiological approaches to clinical work and research should be discarded, or that we should turn the clock back by 80 years. Rather, we believe that any approach to knowledge can be profitably used in its appropriate context. If we bear in mind that the interpersonal relationship is the most proper setting for human understanding, there is no need to fall into the antagonism between objectivity and subjectivity.

In our view, this is a crucial lesson for any psychiatrist, and it is especially important for current residents, who, in an era marked by a lack of psychopathological and humanistic culture (Andreasen, 2007), risk being educated solely through algorithms and putative biological markers. Indeed, key aims of our research are to highlight the importance of a psychiatry that is able to embrace the relational, and implicitly psychotherapeutic, dimension of the clinical encounter, and to emphasize the value of the clinician’s subjective participation within the clinical relationship itself, extending beyond the diagnostic process.

## Data Availability

The raw data supporting the conclusions of this article will be made available by the authors, without undue reservation.
